# The Role of Intestinal Alkaline Phosphatase in Inflammatory Disorders of Gastrointestinal Tract

**DOI:** 10.1155/2017/9074601

**Published:** 2017-02-21

**Authors:** Jan Bilski, Agnieszka Mazur-Bialy, Dagmara Wojcik, Janina Zahradnik-Bilska, Bartosz Brzozowski, Marcin Magierowski, Tomasz Mach, Katarzyna Magierowska, Tomasz Brzozowski

**Affiliations:** ^1^Department of Ergonomics and Exercise Physiology, Faculty of Health Sciences, Jagiellonian University Medical College, Cracow, Poland; ^2^Department of Physiology, Faculty of Medicine, Jagiellonian University Medical College, Cracow, Poland; ^3^Gastroenterology and Hepatology Clinic, The University Hospital, Jagiellonian University Medical College, Cracow, Poland

## Abstract

Over the past few years, the role of intestinal alkaline phosphatase (IAP) as a crucial mucosal defence factor essential for maintaining gut homeostasis has been established. IAP is an important apical brush border enzyme expressed throughout the gastrointestinal tract and secreted both into the intestinal lumen and into the bloodstream. IAP exerts its effects through dephosphorylation of proinflammatory molecules including lipopolysaccharide (LPS), flagellin, and adenosine triphosphate (ATP) released from cells during stressful events. Diminished activity of IAP could increase the risk of disease through changes in the microbiome, intestinal inflammation, and intestinal permeability. Exogenous IAP exerts a protective effect against intestinal and systemic inflammation in a variety of diseases and represents a potential therapeutic agent in diseases driven by gut barrier dysfunction such as IBD. The intestinal protective mechanisms are impaired in IBD patients due to lower synthesis and activity of endogenous IAP, but the pathomechanism of this enzyme deficiency remains unclear. IAP has been safely administered to humans and the human recombinant form of IAP has been developed. This review was designed to provide an update in recent research on the involvement of IAP in intestinal inflammatory processes with focus on IBD in experimental animal models and human patients.

## 1. Introduction

Alkaline phosphatase (AP) is a superfamily of metalloenzymes known to catalyse the hydrolytic removal of phosphate from a variety of molecules [1]. The mammalian AP family consists of several isozymes which can be classified as tissue-nonspecific APs (TNAP) expressed in bone, liver, and kidney and tissue-specific APs, intestinal, placental, and germ cell type of the enzyme [1, 2]. Intestinal alkaline phosphatase (IAP) exhibits its biological activity in alkaline conditions with highest activity at a pH 9.7 [1, 3–5]. IAP is expressed throughout the gastrointestinal tract with highest expression in duodenum and to a much lesser extent in the jejunum, ileum, and colon, but it has also been detected in the stool. Interestingly, the expression of IAP is almost absent in the healthy stomach, but the TNAP isoform was identified at low levels in the healthy colon [6]. IAP present in the apical microvilli of the brush border of enterocytes and secreted into both the intestinal lumen and the bloodstream has been considered as gut mucosal defence factor essential for maintaining gut homeostasis [3–5]. Fawley and Gourlay [7] reviewing data from several animal and human studies have demonstrated that exogenous IAP exerts the protective effect against intestinal and systemic inflammation in a variety of diseases. Furthermore, the human recombinant IAP is at present undergoing phase 2 of clinical trial clearly suggesting an importance of this enzyme in the therapy of lower GI disorders [7]. The IAP is expressed throughout the intestine with the highest expression in the duodenum, whereas its phosphatase activity is highest in the terminal ileum [7]. The IAP has been shown to inhibit the activation of NF-*κ*B and its translocation into the nucleus, thus preventing the expression of proinflammatory cytokines [7].

## 2. Mechanisms of Action of IAP

Among major functions of IAP in GI tract the most important are the regulation of bicarbonate secretion and pH at duodenal surface, the modulation of intestinal long-chain fatty acids (LCFA) absorption, and the detoxification of endotoxin lipopolysaccharide (LPS) leading to local intestinal and systemic anti-inflammatory effects. These actions of IAP are critical for the maintenance of the normal homeostasis of gut microbiota and the inhibition of their translocation across mucosal barrier of the gut [4, 10]. Of those mentioned, the ability of IAP to inactivate LPS seems to be particularly important. LPS is a major component of the outer membrane of Gram-negative bacteria [11] which compose a substantial part of the mammalian intestinal microbiota [2]. LPS could bind to LPS-binding protein (LBP) and the LBP-LPS complex is transferred to membrane-bound or soluble CD14, thereby enabling interactions with toll-like receptors (TLRs) on cell membranes [12]. Upon activation by LPS, toll-like receptor 4 (TLR4) initiates a signalling cascade leading to the release of proinflammatory cytokines as tumour necrosis factor-*α* (TNF-*α*), interleukin- (IL-) 1, IL-6, IL-8, and IL-12 [13]. Endotoxemia resulting from the abundance of LPS in the blood stream can lead to a septic shock, if the immune response is severely pronounced but at lower levels of immunity, it can also stimulate mild chronic inflammatory response associated with chronic inflammatory diseases [14].

These effects of LPS are mainly mediated by the lipid A moiety of LPS, which permits its binding to the toll-like receptor 4 (TLR4) and activate two distinct pathways, namely, myeloid differentiating factor 88- (MyD88-) dependent and MyD88-independent pathway. Initiation of MyD88-dependent pathway could lead to activation of nuclear factor-*κ*B (NF-*κ*B) and release of several proinflammatory cytokines [15].

**Figure 1:**
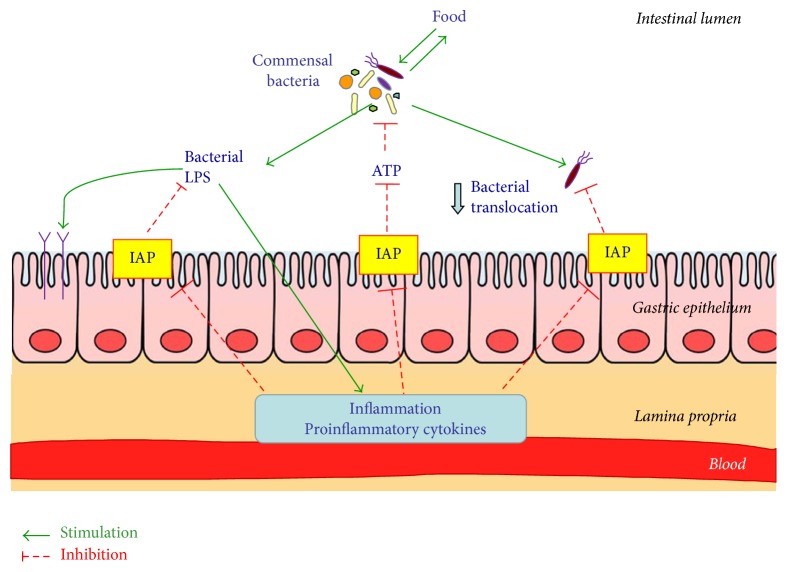
Hypothetical model depicting the mechanism by which a brush border enzyme intestinal alkaline phosphatase (IAP) affects the intestinal microbiota, the release of bacterial LPS-induced inflammation, and the luminal content of ATP inhibiting the commensal bacteria of different origin. Under inflammation, the proinflammatory cytokines can inhibit the content and activity of protective IAP. The IAP can dephosphorylate bacterial LPS which leads to LPS detoxification, thus preventing downstream activation of immunocytes and the subsequent inflammatory responses. The IAP can inhibit luminal ATP by the mechanism involving the ATP dephosphorylation. This enzyme was found to exert an inhibitory effect on the growth and survival of a wide spectrum of bacteria and to prevent bacteria translocation from intestinal lumen into bloodstream. The mechanisms illustrated in this figure which are described in the text were inspired by [8, 9] cited in this review.

The toxic moiety of lipid A in LPS contains two phosphate groups that are essential for its biological action [16]. IAP plays a vital role in promoting mucosal tolerance to gut bacteria via dephosphorylation of LPS and, in general, APs have been shown to remove lipid A phosphate of the LPS [17–19]. The two phosphate groups of the LPS lipid A moiety support stable binding to the receptor complex, and the dephosphorylation of lipid A leads to the amelioration of inflammatory activity of LPS [20]. IAP has been shown to inhibit MyD88-dependent pathway by preventing of the activation of TLR4, thus dampening the activation of NF-*κ*B and ultimately repressing the downstream of TLR-4-dependent and inflammatory cascades [3, 5, 15]. Consequently, the dephosphorylated LPS due to IAP is no longer able to cause TLR4 stimulation and may even block its binding to receptor acting as an antagonist of TLR-4 receptors [4, 10, 21]. The LPS interaction with the IAP has been supported by the fact that the administration of exogenous IAP attenuated LPS-mediated toxicity [22, 23] (Figure 1). Another potential IAP targets could be unmethylated cytosine-guanosine dinucleotides (CpG, a component of bacterial DNA) and flagellin (a protein found in bacterial flagella) both of which known to induce host inflammatory responses [24]. It has been demonstrated that inhibition of endogenous IAP by L-phenylalanine increases serum LPS levels [24].

## 3. IAP and the Intestinal Microbiota

The gastrointestinal tract contains an enormous number of microorganisms, collectively known as the gut microbiota. The intestinal microbiota plays a significant role in maintaining human health and dysbiosis defined as pathological imbalance in the gut microbiota. The alteration in intestinal microbiota has been implicated in the pathogenesis of various diseases including inflammatory bowel disease (IBD), antibiotic-associated diarrhoea (AAD),* Clostridium difficile*-associated disease (CDAD), metabolic syndrome, obesity, and cancer [30, 31].

It has been suggested that IAP can directly regulate maintenance of normal gut microbial homeostasis. Bates et al. [32] proposed that IAP could play an essential role in promoting mucosal tolerance to the resident gut bacteria by preventing LPS-mediated inflammatory response. In IAP-knockout mice, increased bacterial translocation to mesenteric lymph nodes was observed when the intestine was subjected to a ischemic injury [21]. Malo et al. [31] have demonstrated that IAP-KO mice exhibited much less of different types of aerobic and anaerobic bacteria than the wild-type mice and that oral supplementation of IAP reversed this abnormality. They have concluded that IAP is involved in the maintenance of normal gut microbial homeostasis and suggested the therapeutic potential of this enzyme [31]. In another study by the same group of investigators, a mechanism by which IAP may positively regulate the intestinal microbiome was identified, inactivation (dephosphorylation) of phosphorylated nucleotides in the intestinal lumen [8]. They observed that IAP-knockout mice exhibited more ATP in their luminal contents than the wild-type mice [32]. Moreover, the exogenous IAP was able to reverse the ATP-mediated inhibition of bacterial growth [8] (Figure 1). It was suggested that IAP stimulates bacterial growth in the intestinal lumen and luminal ATP inhibits mainly Gram-positive bacteria. This inhibition could be reversed by exogenous IAP, which dephosphorylates free ATP [9]. The administration of exogenous IAP in mice showed a decrease in severity rates of* Salmonella typhimurium* and* Clostridium difficile*-associated disease activity with IAP's ability to rapidly restore the regular gut microbiota [33].

## 4. Diet and IAP

Diet has been regarded as an important factor in many chronic inflammatory diseases such as IBD or metabolic syndrome. That is why an assumption aroused that IAP expression and activity as for other intestinal enzymes can be regulated by diet. For instance, Goldberg et al. [21] have shown that the IAP expression and LPS-dephosphorylating activity were dramatically decreased after two days of fasting in mice maintained at enteral feeding [21]. They suggested that the IAP silencing that occurs with starvation may, at least in part, explain the impaired gut mucosal defence seen in critically ill patients. Clinical studies have documented the protective effects of trophic enteral feeding in patients who were unable to tolerate a normal oral diet, but the underlying mechanism of this barrier dysfunction in these patients has not been so far explained [21]. Some controversy exists on the effect of high-fat diet (HFD) with respect to IAP activity. Alpers et al. [3] have shown an increase in IAP secretion in response to HFD possibly as a consequence of intestinal membrane self-defence protective mechanisms that can oppose deleterious effect resulting from an enhancement in microbial LPS levels [34, 35]. However, in another study, HFD resulted in a downregulation of IAP expression [35]. A possible explanation could be that this downregulation of IAP is due to the enhanced concentration of the fat in HFD diet known to affect the IAP regulation in intestine of these animals [35].

## 5. IAP and Inflammatory Chronic Diseases

Decreased IAP expression has been implicated in many chronic inflammatory diseases such as inflammatory bowel disease (IBD), celiac disease, metabolic syndrome, and obesity.

### 5.1. IBD

Recent evidence indicates that IAP may have a therapeutic role in IBD without the risk of harmful effects associated with currently accepted therapies. IBDs are a heterogeneous group of disorders exhibiting two major forms, Crohn's disease (CD) and ulcerative colitis (UC), characterized by a cyclic nature, alternating between active and quiescent states [36]. The IBD aetiology is still relatively unknown and the current concept is that a combination of environmental agents and a dysfunctional mucosal immune system in genetically susceptible individuals could lead to the development of this disease [37–39]. The incidence rates and prevalence of IBD over the past 50 years have increased remarkably in countries that have adapted a “westernized” lifestyle [40, 41] characterized by serious modifications in dietary habits and decreased physical activity. The composition of the gut microbiota is thought to be a critical factor in the development of IBD and an association between diet and the composition of the human microbiome has been demonstrated [42–45]. Dietary patterns characteristic for industrialized developed countries might promote a dysbiosis in genetically predisposed subjects [45]. In an experimental mouse model, the chronic high-fat diets have altered the composition of gut microbiota leading to a “dysbiotic” microbiome and an increase in the incorporation into chylomicrons of LPS. This effect not only compromised the gut mucosal integrity but also promoted the mucosal entry of pathogenic agents from the intestinal lumen into the bloodstream [46, 47]. Deficiencies in response mechanisms such as IAP activity against bacterial products, in particular LPS, have been shown to be an important factor underlying IBD pathogenesis [48, 49]. Dysbiosis and decreased complexity of the gut microbial ecosystem are common features in IBD patients [50]. IBD patients have a significantly higher TLR4 expression when compared to healthy controls [51, 52]. Cario and Podolsky have collected small intestinal and colonic biopsy specimens from IBD patients and observed that TLR4 was strongly upregulated in intestinal epithelium of UC and CD patients [51]. Similarly, the increased levels of TLR4 in the inflamed colonic mucosa of children with IBD were observed. Interestingly, the TLR4 mRNA levels were similar to controls in the noninflamed colonic mucosa of children with IBD [52].

Furthermore, UC or CD patients have demonstrated a low IAP activity compared with normal healthy controls [19, 53]. Consequently, the impairment of the intestinal protective mechanism observed in patients with IBD has been attributed to the lower intestinal synthesis and activity of endogenous IAP, but the pathomechanism of this disorder with the respect to IAP deficiency has not been fully explored [19].

#### 5.1.1. Animal Studies

Several studies have examined the role of IAP in experimental colitis (Table 1). In IAP-knockout (IAP-KO) mice with ischemia-induced intestinal barrier dysfunction, the increased intestinal inflammation was observed possibly due to promotion of the bacterial translocation through intestinal barrier in these animals as compared to wild-type mice [21]. In another study, Martínez-Moya et al. [6] have shown that intrarectal IAP administration prevented bacterial translocation in different models of colitis. Moreover, although the IAP-KO mice did not exhibit spontaneous colitis, they have been shown to be more susceptible to experimental colitis [54]. Tuin et al. [19] studied the efficacy of orally administrated acid resistant IAP tablets on dextran sodium sulfate- (DSS-) induced experimental colitis in rats. They revealed a significant reduction of DSS-induced inflammation with IAP therapy [19].

A similar observation was made in a mouse model of DSS-induced colitis by another group of investigators [25]. They found the loss in body weight being significantly less in IAP-treated mice and accompanied with reduced colon damage as manifested in their study by a histological index of disease activity, for example, the crypt and goblet cell loss, intestinal oedema, and less severe neutrophils infiltration of intestinal wall [25].

Ramasamy et al. [26] evaluated the potential therapeutic role for orally administered IAP in two independent mouse models of chronic colitis, namely, DSS-induced mouse colitis model in both wild-type mice and IAP-knockout mice, and the irradiation affected mice deficient in Wiskott-Aldrich Syndrome Protein (WASP) colitis. They found that orally administered IAP significantly attenuated inflammation in both IAP-knockout and wild-type mice in this chronic colitis model [26]. Lee et al. [27] have employed the piroxicam-induced IL-10 knockout mouse model and observed the significant reduction in the severity of experimental colitis after oral administration of IAP. These results strongly suggest that the lower activity of endogenous IAP is associated with an increased severity of intestinal injury in experimental colitis and this deleterious effect is reversed by supplementation with exogenously administrated IAP.

#### 5.1.2. Human Studies

In the human trial [29], the administration of IAP daily over a 7-day course to patients with UC was associated with a short-term improvement in disease activity scores, and clinical beneficial effects were observed within 21 days and associated with reductions in C-reactive protein and stool calprotectin. It is of interest that the treatment with exogenous IAP was well tolerated without signs of toxicity in these patients [29], as shown in Table 2.

The hypertrophied mesenteric adipose tissue could be a major contributor of the increased circulating proinflammatory cytokines in IBD and undoubtedly play a role in the pathogenesis and activity of this disease [55–58]. Because of the formation of so-called “creeping fat,” this increase in proinflammatory cytokines can be, at least in part, caused by LPS [57, 59, 60], but the IAP administration is believed to reduce this process. Moreover, the chronic exercise could also contribute to the amelioration of proinflammatory cytokines by stimulating secretion of vasodilatory myokines from active muscle units, factors that were shown to influence muscle-fat crosstalk [61–63].

Several lines of evidence indicate that increased intestinal permeability may have crucial role in IBD pathogenesis [64–67]. Paracellular permeability is regulated by junctional complexes: on the apical aspect, cells are linked by tight junctions (TJ) and adherents junctions as well as by desmosomes at the basolateral compartment. Molecular components of tight junctions include integral membrane proteins: occludins, claudins, and junctional adhesion molecules (JAM) as well their scaffolding proteins (zonula occludens (ZO) proteins) [68]. A reduced expression and redistribution of occludins, claudins, and JAM have been reported in IBD patients [69–71]. Guo et al. [72] demonstrated that LPS causes an increase in intestinal tight-junction permeability in vitro and in vivo via an intracellular mechanism involving TLR-4-dependent upregulation of CD14 membrane expression. Liu et al. [73] have studied the relationship between IAP deficiency and IAP overproduction and their effect on tight-junction protein (TJP) levels and function. They have revealed that the pretreatment with IAP prevented the decline in junctional protein expression, and this effect was proposed to explain the mechanism of abrogation of the LPS-induced barrier dysfunction in vitro. Furthermore, the administration of IAP effectively strengthens the barrier function by a preservation of its integrity as well as prevented the LPS-induced potential alteration in the localization and assembly of TJ. These observations suggest a crucial role for IAP in the maintenance of epithelial homeostasis and barrier function. Liu et al. [73] concluded that IAP is a chief regulator of gut mucosal permeability and may act, at least in part, by improving TJP levels and localization.

In excellent review, Lallès [10] suggested possible opposing effects of two AP isoforms in IBD. Based on clinical studies [19, 28, 29] and animal experiments [6, 19, 25, 26, 74, 75], Lallès [10] observed that IAP is reduced, but TNAP is upregulated in IBD. An increased TNAP seems to originate from both colonic epithelial cells and neutrophils. Lallès [10] suggested that colonic upregulation of TNAP under inflammatory conditions could represent a protective intestinal adaptation against oxidative stress and progression of inflammation.

### 5.2. Other Chronic Diseases

The role of IAP deficiency in the pathogenesis of metabolic syndrome has been especially well studied. Metabolic syndrome is a cluster of conditions: central obesity, elevated blood pressure, elevated insulin resistance, dyslipidaemia, and fatty liver. Metabolic syndrome leads to type 2 diabetes, atherosclerosis, and nonalcoholic fatty liver disease [76, 77]. Previous studies revealed that the high-fat diet (HFD) modulates gut microbiota and increases the plasma concentration of LPS and triggers body weight gain and diabetes [78, 79]. Moreover, endotoxemia has been suggested to play a crucial role in pathogenesis of metabolic syndrome [78, 79].

An essential role of LPS was confirmed by the observation that mice lacking TLR4, the receptor for LPS, are resistant to HFD-induced inflammation, obesity, and insulin resistance [80]. Mice who were made deficient of IAP exhibited an increased intestinal permeability and high levels of LPS, obesity, the elevated blood glucose and insulin resistance [34]. The combined high-fat diet with IAP prevented completely the development of metabolic syndrome [34]. In another study, the antibiotic treatment early in life rendered mice more susceptible to metabolic syndrome in adulthood, but coadministration of IAP with antibiotic prevented completely this susceptibility to metabolic syndrome [81].

The intestinal barrier integrity is impaired in celiac disease. This has been referred to a decrease in the IAP expression and activity in celiac paediatric patients [81]. Furthermore, this intestinal impairment was particularly pronounced in severe cases of the disease [82]. IAP expression was normalised in children on gluten free diet. The same group also found our increased mRNA and protein levels of TLR4 in the duodenal mucosa of newly diagnosed celiac paediatric patients [83]. They have suggested that uncontrolled activation of TLR4 by LPS may induce loss of mucosal function due to an induction of a proinflammatory cascade [82].

Potential protective role of IAP was suggested in a number of other diseases and experimental animal models translating basic research to the clinical settings. Exogenous IAP had also beneficial effects in experimental model of necrotizing enterocolitis (NEC) and was suggested to be a valuable adjunctive treatment in NEC [18]. Ebrahimi et al. [84] have demonstrated that peritoneal irrigation with IAP potently enhanced survival in a mouse model of peritonitis. In recent paper, Wang et al. [85] have shown a therapeutic effect of exogenous IAP in mouse model of peritonitis, as manifested by the inhibition of intestinal permeability and bacterial translocation. Exogenous IAP had also protective action in mice with antibiotic-associated diarrhoea [86]. Furthermore, the significant fall in the activity of IAP has been documented in duodenal biopsies taken from patients with cystic fibrosis (CF) [87, 88]. Also in CF mouse model, the decreased levels of IAP and increased intestinal permeability have been observed [89].

## 6. IAP and Septic Shock

Sepsis is a predominant cause of acute kidney injury (AKI) in the intensive care unit [90] which is associated with a high mortality and an increased risk of developing chronic kidney failure in survivors. Two double-blind, randomized, placebo-controlled phase II clinical trials have demonstrated a beneficial renal effects of bovine IAP administration in critically ill patients with sepsis-associated AKI [91, 92]. These protective effects of IAP were attributed to dephosphorylation of LPS and the rise in extracellular ATP [93, 94]. Administration of bovine-derived material in humans is limited and represents many drawbacks. Therefore, a human recombinant AP (recAP) has been recently developed [95] and its protective effects have been confirmed in studies in vitro and in vivo in rat model of LPS-induced AKI [96]. In a randomized, double-blind, placebo-controlled, phase I trial with healthy volunteers, the pharmacokinetics, safety and tolerability of recAP was investigated and these results failed to raise safety concerns [97].

## 7. Conclusion

IAP role as key regulator of inflammation, infection, and gut microbiota mainly via dephosphorylation of LPS has been established. Diminished activity of IAP could increase the risk of disease through changes in the microbiome, increased intestinal inflammation, and intestinal permeability. In light of accumulated evidence presented in this review and the fact that the human recombinant form of IAP (recAP) has been developed and this recombinant form of IAP has been safely administered to humans without any adverse effects, we conclude that IAP may represent a potential therapeutic agent to improve outcomes of inflammatory diseases driven by gut barrier dysfunction such as IBD. The discovery of recombinant form of IAP raises a hope that clinical studies in the future can shed more light on the intriguing opportunity of a therapeutic strategy concerning the efficacy of this enzyme in treatment of human diseases especially to prevent dysbiosis and intestinal and systemic inflammation frequently associated with lower GI tract disorders such as IBD, IBS, and* Clostridium difficile* infection.

## Figures and Tables

**Table 1:** Characteristics of animal studies examining potential role of IAP in experimental colitis.

Reference	Study type	Study outcome
Goldberg et al., 2008 [21]	IAP-knockout mice (C57BL/6 background)	In mice with intestinal barrier dysfunction induced by ischemia the IAP-knockout mice had increased severity of intestinal inflammation and increased bacterial translocation as compared to wild-type mice.

Martínez-Moya et al. 2012 [6]	Wistar rats	IAP was given to rats by the oral or intrarectal route and have beneficial effects on experimental trinitrobenzene sulfonic acid (TNBS) and dextran sulfate sodium (DSS) model of rat colitis, which includes protection against bacterial translocation. Treatment with intrarectal IAP was more effective than oral route.

Tuin et al., 2009 [19]	Sprague-Dawley Rats	Orally administered IAP caused a significant reduction of inflammation in rat model of DSS -induced colitis in rats.

Bol-Schoenmakers et al., 2010 [25]	C57BL/6J mice	In DSS induced colitis, the oral IAP administration exerts a beneficial effect against severe intestinal epithelial damage. Rectal administration of LPS into a moderate inflamed colon did not aggravate inflammation.

Ramasamy et al., 2011 [26]	IAP-KO and WT C57BL/6 mice WASP-KO (129 SvEv background) mice	In two independent mouse models of chronic colitis: DSS-induced mouse colitis model in wild type mice and IAP knock out mice and the irradiation induced WASP-KO colitis, orally administered IAP significantly attenuated inflammation in both, IAP-knockout and wild-type mice in the chronic colitis model.

Lee et al., 2014 [27]	IL-10−/− mice (C57BL/6 background)	In piroxicam-induced IL-10 knockout mice, the significant reduction of experimental colitis severity was observed after oral administration of IAP.

**Table 2:** Characteristics of studies examining the role of IAP in human IBD.

Reference	Sample	Outcome
Molnár et al., 2012 [28]	CD, 10 children (7 boys, 3 girls) UC, 5 children (3 boys, 2 girls) Control, 10 children (5 boys, 5 girls;)	Significantly decreased IAP levels in inflamed mucosa in children with IBD.

Tuin et al., 2009 [19]	CD, 10 (3 males, 7 females) UC, 14 (9 males, 5 females) Control, 14 (8 males, 6 females)	Reduced IAP mRNA expression in inflamed mucosa in adults with UC and CD.

Lukas et al., 2010 [29]	UC, 23 females	Improvement in disease activity scores, with clinical effects being observed within 21 days and associated with reductions in C-reactive protein and stool calprotectin.
